# Modelling quiescence exit of neural stem cells reveals a FOXG1-FOXO6 axis

**DOI:** 10.1242/dmm.052005

**Published:** 2024-11-29

**Authors:** Kirsty M. Ferguson, Carla Blin, Claudia Garcia-Diaz, Harry Bulstrode, Raul Bardini Bressan, Katrina McCarten, Steven M. Pollard

**Affiliations:** Centre for Regenerative Medicine, Institute for Regeneration and Repair and Edinburgh Cancer Research UK Centre, The University of Edinburgh, Edinburgh EH16 4UU, UK

**Keywords:** Neural stem cell, Quiescence, FOXG1, FOXO6, Pak1, Glioblastoma

## Abstract

The molecular mechanisms controlling the balance of quiescence and proliferation in adult neural stem cells (NSCs) are often deregulated in brain cancers such as glioblastoma multiforme (GBM). Previously, we reported that FOXG1, a forebrain-restricted neurodevelopmental transcription factor, is frequently upregulated in glioblastoma stem cells (GSCs) and limits the effects of cytostatic pathways, in part by repression of the tumour suppressor *Foxo3*. Here, we show that increased FOXG1 upregulates *Foxo6*, a more recently discovered FOXO family member with potential oncogenic functions. Although genetic ablation of *Foxo6* in proliferating NSCs had no effect on the cell cycle or entry into quiescence, we found that *Foxo6*-null NSCs could no longer efficiently exit quiescence following FOXG1 elevation. Increased *Foxo6* resulted in the formation of large acidic vacuoles, reminiscent of Pak1-regulated macropinocytosis. Consistently, Pak1 expression was upregulated by FOXG1 overexpression and downregulated upon FOXO6 loss in proliferative NSCs. These data suggest a pro-oncogenic role for FOXO6, downstream of GBM-associated elevated FOXG1, in controlling quiescence exit, and shed light on the potential functions of this underexplored FOXO family member.

## Teaser

**Summary:** This study finds a pro-oncogenic role for FOXO6, downstream of glioblastoma-associated elevated FOXG1, in controlling quiescence exit of mouse neural stem cells.

## INTRODUCTION

Stem cell fate is orchestrated by gene regulatory networks of lineage-specific master regulatory transcription factors (Graf and Enver, 2009). Just as tissues rely on these factors for proper development, cancers can subvert developmental networks to impose a stem cell-like state that underpins tumour growth (Roy and Hebrok, 2015; Huilgol et al., 2019). Glioblastoma multiforme (GBM), the most common and aggressive primary adult brain cancer, is driven by glioblastoma stem cells (GSCs) that display neural stem cell (NSC) characteristics (Singh et al., 2003; Pollard et al., 2009; Richards et al., 2021). GSCs frequently overexpress key neurodevelopmental transcription factors to drive their self-renewal and restrict differentiation (Engström et al., 2012; Suva et al., 2014; Carén et al., 2015; Singh et al., 2017). One such factor is the Forkhead box transcription factor FOXG1. FOXG1 has important roles in telencephalon development and *in vitro* reprogramming (Xuan et al., 1995; Lujan et al., 2012; Bulstrode et al., 2017). *FOXG1* is one of the most consistently overexpressed genes across GBM molecular subtypes, and high levels are associated with adverse outcomes (Engström et al., 2012; Verginelli et al., 2013; Robertson et al., 2015; Wang et al., 2018). Understanding the molecular mechanisms through which FOXG1 operates in NSCs and GSCs is therefore of great interest.

Both GSCs and genetically normal NSCs are known to be heterogeneous with regards to cell cycle status, with cells spanning a continuum from dormant to quiescent and proliferative states (non-cycling, slow-cycling and fast cycling, respectively) (Codega et al., 2014; Dulken et al., 2017; Marqués-Torrejón et al., 2021; Llorens-Bobadilla et al., 2015). Quiescent GSCs evade anti-mitotic therapies and hijack NSC-like properties to drive tumour re-growth (Deleyrolle et al., 2011; Chen et al., 2012; Ishii et al., 2016). Thus, understanding the mechanisms controlling GSC quiescence will be important for the design of rational therapeutic strategies that might suppress patient relapse.

NSCs expanded in culture have overlapping gene regulatory networks with GBMs and provide a genetically tractable experimental *in vitro* model that has been useful in delineating the pathways controlling GSC quiescence (Ying et al., 2003; Conti et al., 2005; Sun et al., 2008; Pollard et al., 2009; Carén et al., 2015; Bulstrode et al., 2017; Bressan et al., 2021; Marqués-Torrejón et al., 2021). Bone-morphogenetic protein 4 (BMP4) induces quiescence of NSCs *in vitro* and *in vivo* (Mira et al., 2007; Sun et al., 2011; Bond et al., 2012; Martynoga et al., 2013; Marqués-Torrejón et al., 2021), while the mitogens EGF and FGF-2 stimulate proliferation. Previously, we demonstrated that overexpression of the GBM-associated master regulators FOXG1 and SOX2 drives quiescent mouse NSCs into a proliferative radial glia-like state (Bulstrode et al., 2017) and induces transcriptional changes at many key cell cycle and epigenetic regulators. In particular, *Foxo3*, which induces quiescence and prevents premature NSC differentiation, is directly repressed by FOXG1 (Renault et al., 2009; Bulstrode et al., 2017). FOXG1 is therefore an important regulator of quiescence in NSCs and GSCs. Using NSCs to determine the genes and pathways operating downstream of elevated FOXG1 will therefore help our understanding of normal NSC development, adult NSC homeostasis and GBM biology.

The FOXO family are key downstream effectors of PI3K-Akt signalling, controlling genes governing diverse cellular processes including proliferation, metabolism, differentiation and apoptosis. Although FOXO factors can have context-dependent roles in supporting cellular resilience, they are most well known for their tumour-suppressive functions in tissue homeostasis, ageing and cancer (Dansen and Burgering, 2008; Hornsveld et al., 2018). FOXO1/3/4 are broadly expressed during development and adulthood, and, although discrete roles have been identified, they appear to regulate common target genes *in vitro* with likely significant redundancies (Paik et al., 2007).

FOXO6 is the most recently identified FOXO member. It was initially reported to be expressed mainly within the central nervous system of adult mammals (Hoekman et al., 2006), but may also have roles in other tissues such as liver and muscle (Kim et al., 2011). Compared to FOXO1/3/4, it has several unique molecular characteristics: FOXO6 has a low sequence homology (∼30%) to other FOXO factors, lacks one of three consensus PKB phosphorylation sites, and the presence of a nuclear export signal is debated (Kim et al., 2013). Unlike other FOXO members, FOXO6 does not undergo complete nucleocytoplasmic shuttling in response to PI3K-Akt-mediated phosphorylation (Jacobs et al., 2003; van der Heide et al., 2005). These features suggest a distinct cellular function. Indeed, in several cancers, FOXO6 is elevated and has oncogenic roles, triggering increased proliferation and progression (Qinyu et al., 2013; Rothenberg et al., 2015; Wang et al., 2017; Lallemand et al., 2018).

Here, we demonstrate that FOXO6 is transcriptionally activated downstream of elevated FOXG1 in mouse NSCs and GSCs and is necessary for FOXG1-driven exit from quiescence. Following forced expression of FOXO6, we observed stimulation of macropinocytosis, a cellular process involved in nutrient uptake that requires Pak1-regulated actin cytoskeleton remodelling. Gain- and loss-of-function mechanistic studies demonstrated that Pak1 is upregulated by FOXG1 overexpression and downregulated upon FOXO6 loss in proliferative NSCs. Altogether, these data suggest a pro-oncogenic role for FOXO6, downstream of GBM-associated elevated FOXG1, in the regulatory transitions that must be initiated as cells move from quiescence to proliferation.

## RESULTS

### FOXG1 transcriptionally activates *Foxo6* in mouse NSCs and GSCs

Previously, we found that overexpression of the master regulators FOXG1 and SOX2 supports cell cycle re-entry of quiescent mouse NSCs. Chromatin immunoprecipitation followed by sequencing (ChIP-seq) and RNA-sequencing (RNA-seq) data identified *Foxo6* as a strong candidate FOXG1/SOX2-regulated target gene (Bulstrode et al., 2017). Here, we hypothesise that, in contrast to FOXO3, FOXO6 has unique roles in supporting proliferation. To investigate the effect of elevated FOXG1 on *Foxo6* expression – thereby mimicking the increased levels seen in GBMs – we used clonal adult mouse NSC lines, derived from adult subventricular zone (SVZ), harbouring a doxycycline (Dox)-inducible *FOXG1-V5* construct, as reported previously (Bulstrode et al., 2017).

**Fig. 1.**
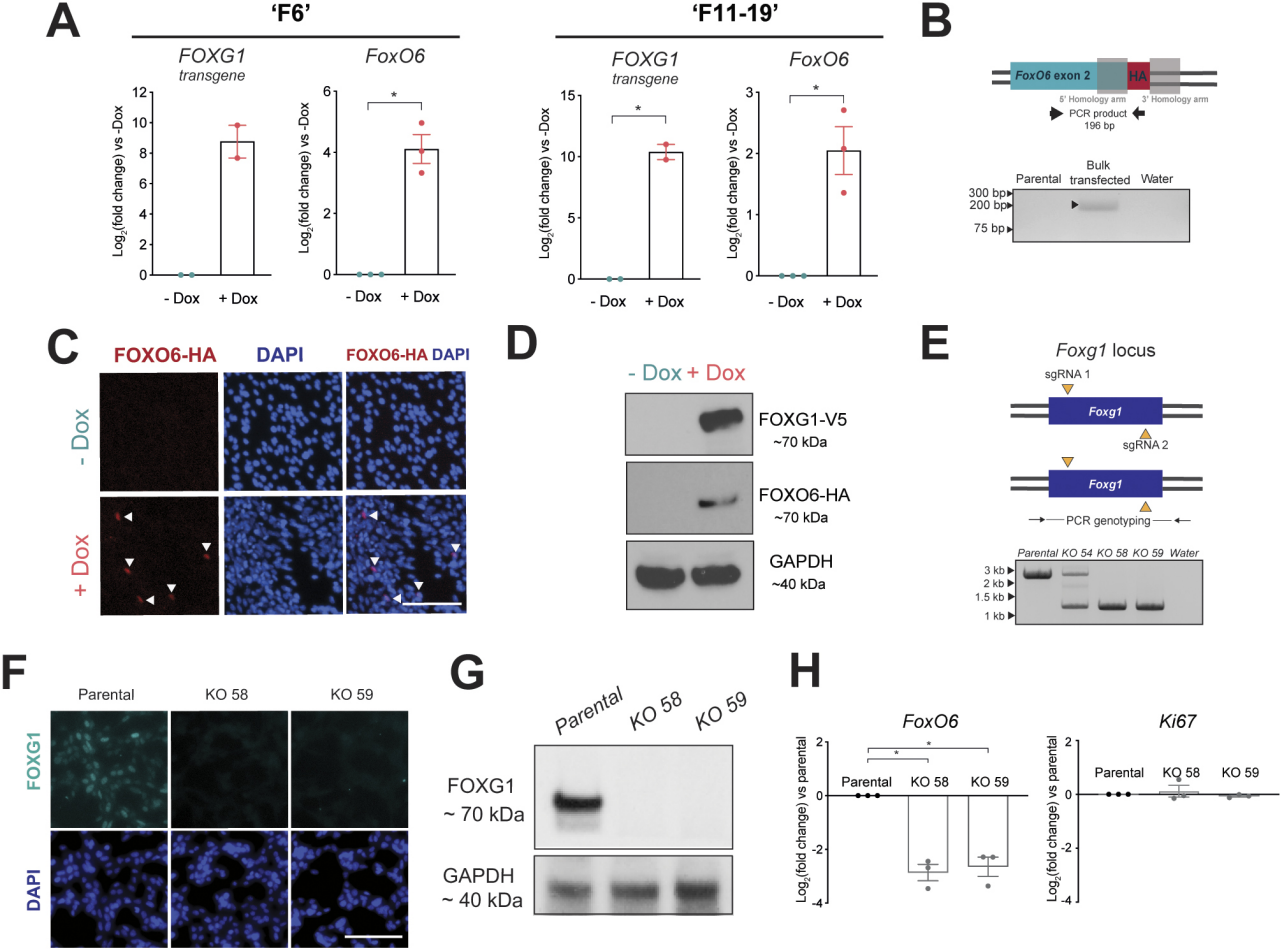
**Elevated FOXG1 transcriptionally activates *Foxo6* in mouse neural stem cells (NSCs) and glioblastoma stem cells.** (A) Quantitative real-time PCR (qRT-PCR) analysis of *FOXG1* transgene and endogenous *Foxo6* expression in two independent adult mouse NSC lines [F6 (left) and F11-19 (right)] with doxycycline (Dox)-inducible *FOXG1-V5* expression grown in NSC medium with or without Dox for 24 h. Expression shown relative to −Dox [in which log_2_(fold change)=0]. Mean±s.e.m. The *y*-axis shows log_2_(fold change). *n*=2/3 independent experiments for F6 and F11-19, respectively. Each data point shows the mean of one experiment performed in technical duplicates. Two-tailed one-sample *t*-test. *P≤0.05. (B) Schematic of homology-directed repair-mediated knock-in of a haemagglutinin (HA) epitope tag at the 3′ end of the last *Foxo6* coding exon in F6 cells. PCR genotyping of the bulk transfected F6 cell population revealed a 196 bp product, indicating the presence of cells with insertion of the HA tag at the 3′ end of *Foxo6*. (C) Wide-field immunofluorescent images following immunocytochemistry (ICC) of FOXO6-HA (red) and DAPI (blue) in the tagged F6 NSCs following Dox addition for 4 days in EGF/FGF-2. Scale bar: 100 μm. (D) Western blot analysis of FOXG1-V5 and FOXO6-HA protein expression in tagged F6 NSCs following Dox addition for 4 days in EGF/FGF-2. GAPDH was used as a loading control. (E) Experimental strategy for *Foxg1* deletion in IENS-GFP cells. Yellow triangles show the target sites of the sgRNAs at either the 5′ or 3′ end of the coding exon. PCR genotyping of parental IENS-GFP cells and *Foxg1* knockout (KO) clonal cell lines (KO 58 and KO 59). Wild-type PCR product, ∼2.6 kb; KO PCR product, ∼1.3 kb. (F) ICC confirms the loss of FOXG1 protein expression in IENS-GFP *Foxg1* KO clonal lines (KO 58 and KO 59). Scale bar: 100 μm. (G) Western blot analysis confirming the loss of FOXG1 protein in the two independent IENS-GFP KO clonal lines (KO 58 and KO 59). GAPDH was used as a loading control. (H) qRT-PCR analysis of *Foxo6* and *Ki67* expression in IENS-GFP *Foxg1* KO clonal lines, compared to parental IENS-GFP [in which log_2_(fold change)=0]. Mean±s.e.m., *n*=3 independent experiments. Each data point shows the mean of one experiment performed in technical duplicates. Two-tailed one-sample *t*-test. *P≤0.05.

Elevated FOXG1-V5 was found to significantly increase *Foxo6* expression in proliferating NSCs (Fig. 1A). After 24 h, *Foxo6* levels increased by ∼17-fold and ∼4-fold in two independent clonal NSC lines, termed F6 and F11-19, respectively [shown as log_2_(fold change)]. To circumvent a lack of FOXO6-specific antibodies, a haemagglutinin (HA) tag was inserted using CRISPR/Cas9-mediated homologous recombination at the 3′ end of *Foxo6* in F6 cells (Fig. 1B). In agreement with mRNA upregulation, we observed clear induction of FOXO6 protein in response to elevated FOXG1 (+Dox) (Fig. 1C,D). These data indicate that *Foxo6* mRNA and protein expression are activated downstream of FOXG1 in normal NSCs.

We next explored FOXO6 levels in a mouse GBM model cell line (IENS), which expresses FOXG1 at higher levels than in mouse NSCs (Bulstrode et al., 2017). We tested whether *Foxg1* ablation affected *Foxo6* expression. Following CRISPR/Cas9-mediated bi-allelic deletion of *Foxg1* in IENS, a significant decrease (6- to 7-fold, or 84-86%) in *Foxo6* expression was observed in two independent clonal cell lines (Fig. 1E-H). No proliferative deficit was observed upon *Foxg1* deletion, indicated by *Ki67* (also known as *Mki67*) expression, in fitting with our previous findings (Bulstrode et al., 2017) that FOXG1 is dispensable for continued NSC or GSC proliferation *in vitro* (Fig. 1H). Elevated FOXG1 is therefore necessary for FOXO6 expression in GSCs and is sufficient to induce increased FOXO6 expression in NSCs.

### FOXG1 induction of *Foxo6* occurs early during the exit of NSCs from quiescence

**Fig. 2.**
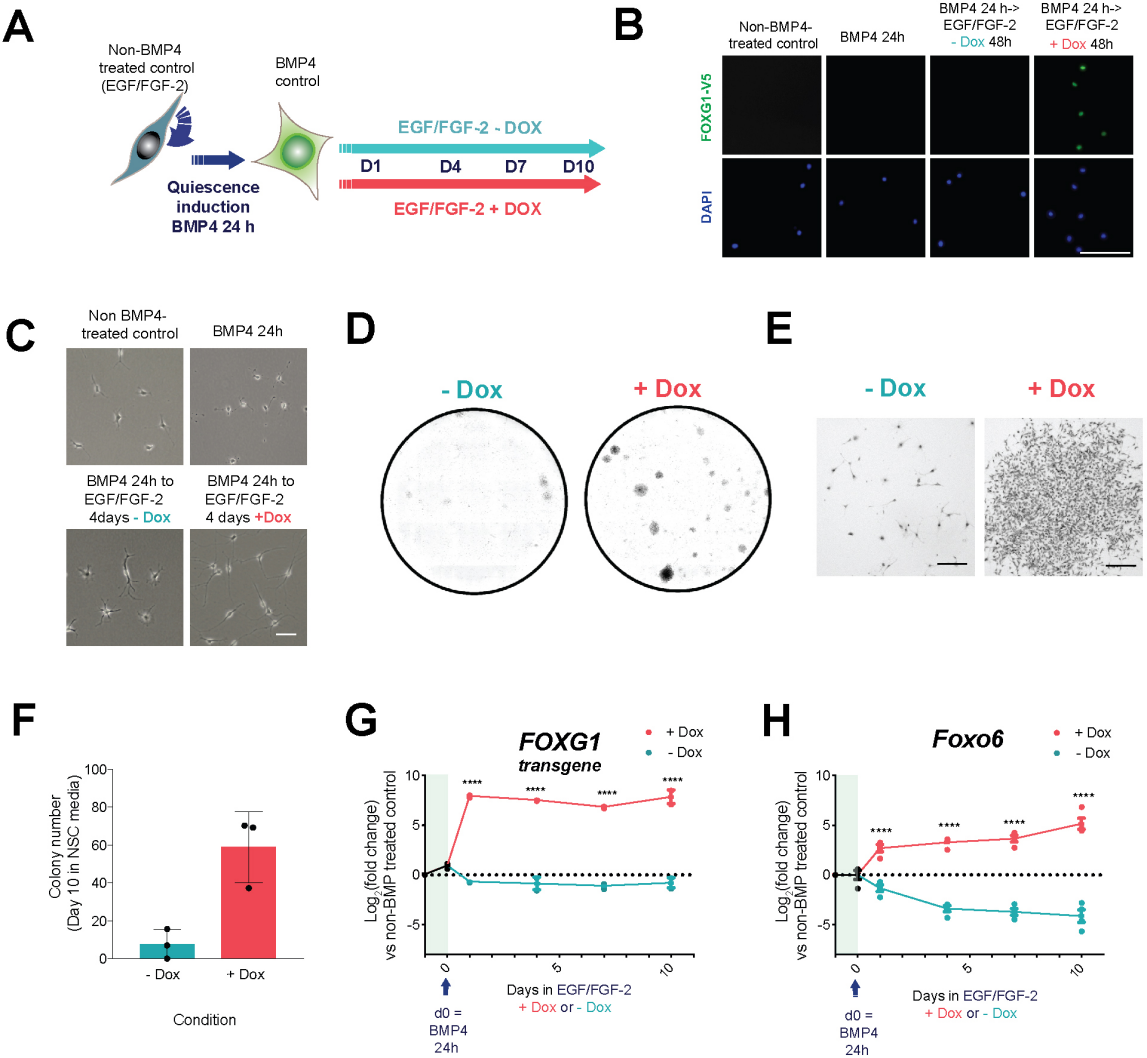
**FOXG1 induces *Foxo6* during quiescent NSC reactivation.** (A) Schematic of the experimental design for assessing FOXG1-induced reactivation of quiescent NSCs and associated changes in gene expression, using clonal F6 adult mouse NSC line with Dox-inducible *FOXG1-V5* expression. Non-BMP4-treated control=cells in NSC medium with EGF/FGF-2. (B) ICC for V5, confirming FOXG1-V5 expression upon Dox addition (scale bar: 100 μm). (C) Representative phase-contrast images showing changes in cell morphology upon addition of Dox (scale bar: 100 μm). (D) Colony formation after 24 h BMP4 treatment followed by 10 days in EGF/FGF-2 with or without Dox. Representative images shown of wells stained with Methylene Blue and imaged on a bright-field microscope. *n*=3 independent experiments. (E) Higher-magnification phase-contrast images of representative colonies after 24 h BMP4 treatment and 10 days in EGF/FGF-2 with or without Dox as in D (scale bars: 200 μm). (F) Number of colonies formed after 24 h BMP4 treatment and 10 days in EGF/FGF-2 with or without Dox. Mean±s.d., *n*=3 independent experiments. Each data point shows the mean of one experiment performed in technical triplicates. (G,H) qRT-PCR analysis of human *FOXG1* transgene (G) and *Foxo6* (H) expression during the reactivation time course. Expression shown relative to non-BMP4-treated (EGF/FGF-2) control [in which log_2_(fold change)=0 (dotted line)]. The *y*-axis shows log_2_(fold change). Day (d)0=expression after 24 h BMP4 treatment. Mean±s.e.m., *n*=2 (*FOXG1*) or 4 (*Foxo6*). Each data point shows the mean of one experiment performed in technical duplicates. Two-way ANOVA with Sidak correction. ****P≤0.0001.

We next assessed *Foxo6* levels during the early phases of NSC exit from quiescence following FOXG1 overexpression. We used quiescence conditions previously shown to induce cell cycle exit, downregulation of NSC markers and upregulation of quiescent marker expression – namely, treatment with BMP4 at low density for 24 h (Fig. 2A) (Bulstrode et al., 2017). Following exchange of BMP4 for culture medium with EGF and FGF-2, FOXG1 induction (+Dox) drove cells to re-enter the cell cycle and form NSC-like colonies (Fig. 2B-F). As expected, we found that Dox addition induced a 235-fold upregulation in *FOXG1* expression by 24 h compared to that in the non-BMP-treated control (EGF/FGF-2) [Fig. 2G, shown as log_2_(fold change)]. *Foxo6* was markedly upregulated at these early timepoints, prior to any visible proliferative response, with a ∼6.5-fold upregulation by 24 h compared to that in the non-BMP4-treated control, ∼16-fold higher than without Dox (Fig. 2H). Increased FOXO6 expression is therefore an early part of the response to FOXG1 in the transition from quiescence to proliferation, consistent with it being an important functional downstream effector.

### FOXG1-induced reactivation of quiescent NSCs is significantly impaired upon *Foxo6* loss

**Fig. 3.**
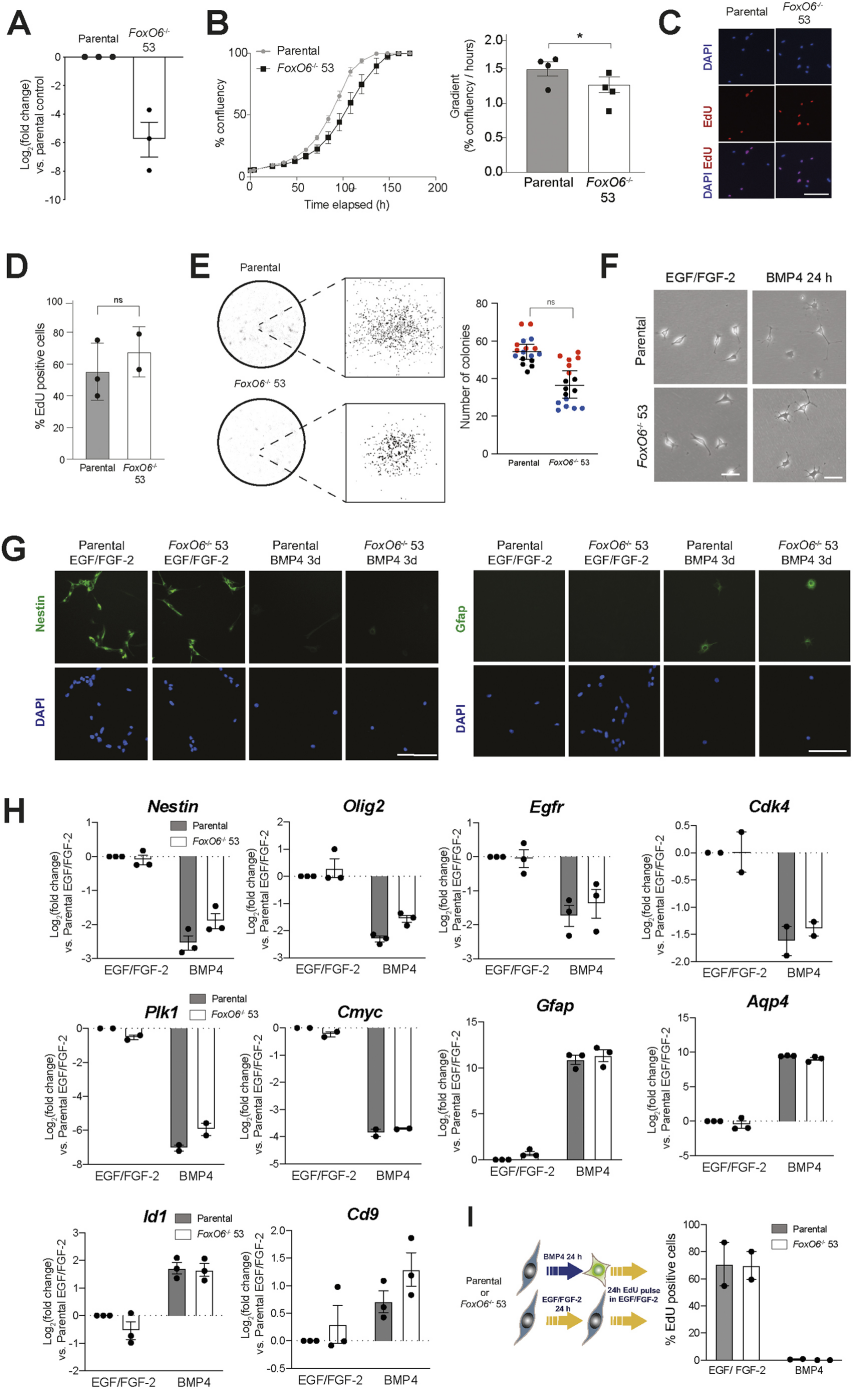
**FOXO6 is not required for continued NSC proliferation or response to BMP4.** (A) qRT-PCR analysis of *Foxo6* mRNA levels in *Foxo6* KO clonal cell line 53, compared to ANS4 parental cells [in which log2(fold change)=0]. Expression values were normalised to *Gapdh*. The *y*-axis represents log_2_(fold change). Mean±s.e.m., *n*=3 independent experiments. Each data point shows the mean of one experiment, performed in technical duplicates. (B) Left: growth curve analysis of parental and *Foxo6* KO 53 clonal cells in EGF/FGF-2. Mean±s.d., *n*=3 technical replicates. Representative of *n*=4 independent experiments. Right: graph showing the gradient of the linear portion of the logistic growth curve (%/h). Mean±s.e.m., *n*=4 independent experiments. Two-tailed paired Student's *t*-test. *P≤0.05. (C) 5-Ethynyl 2′-deoxyuridine (EdU) incorporation assay (24 h pulse) in EGF/FGF-2 for parental and FOXO6 KO 53 cells. Representative fluorescent images of EdU incorporation after 24 h pulse. Scale bar: 100 μm. (D) EdU incorporation assay (24 h pulse) in EGF/FGF-2 for parental and FOXO6 KO 53 cells. Plot shows mean±s.e.m., *n*=3 independent experiments. Each data point shows the mean of one experiment performed in technical triplicates. Two-tailed paired Student's *t*-test. ns, not significant. (E) Left: brightfield images of colony formation by parental or FOXO6 KO 53 cells 10 days after plating at low density in NSC medium (EGF/FGF-2). Plates stained with Methylene Blue. Right: quantification of colony formation in parental or FOXO6 KO 53 cells. Each dot represents one technical replicate (*n*=5/6). Mean±s.e.m., *n*=3 biological replicates, coloured in red, blue or black. Two-tailed paired Student's *t*-test. ns, not significant. (F) Representative phase-contrast images showing morphology of ANS4 and *Foxo6* KO 53 in EGF/FGF-2 medium and after 24 h BMP4 treatment. Scale bar: 25 μm. (G) ICC analysis of NES and GFAP expression in ANS4 parental and FOXO6 KO 53 cells in EGF/FGF-2 or after 3 days’ BMP4 treatment at low density. Scale bars: 100 μm. (H) qRT-PCR analysis of NSC (*Nes*, *Olig2*, *Egfr*), cell cycle (*Plk1*, *Cdk4*, *cMyc*) and astrocyte/quiescence (*Gfap*, *Aqp4*, *Id1*, *Cd9*) markers, in parental and FOXO6 KO 53 cells in EGF/FGF-2 and after 24 h BMP4 treatment. Expression shown relative to parental in EGF/FGF-2 [in which log_2_(fold change)=0]. Mean±s.e.m., *n*=2/3 independent experiments. Each data point shows the mean of one experiment performed in technical duplicates. (I) Left: schematic of the experimental design for determining EdU incorporation after treatment with BMP4 or EGF/FGF-2 for 24 h, followed by a 24 h EdU pulse in EGF/FGF-2. Right: quantification of EdU-positive cells in EGF/FGF-2 and after 24 h BMP4 treatment. Mean±s.e.m., *n*=2 independent experiments. Each data point shows the mean one experiment, performed in technical triplicates.

To assess whether *Foxo6* was required for FOXG1-induced quiescence exit, CRISPR/Cas9 was used to generate clonal adult mouse NSC lines with bi-allelic deletion of the first *Foxo6* coding exon (Fig. S1A,B). Quantitative real-time PCR (qRT-PCR) analysis confirmed loss of *Foxo6*, with a >98% decrease in expression following CRISPR treatment compared to that in untargeted parental cells (Fig. 3A).

*Foxo6*^−/−^ clonal cells displayed a bipolar phase-bright morphology characteristic of proliferative NSCs (Fig. 3F). Although confluence analysis suggested a marginally reduced growth compared to that of parental cells, 5-ethynyl 2′-deoxyuridine (EdU) incorporation did not indicate any significant changes in proliferation (Fig. 3B-D; Fig. S3B-D). Furthermore, *Foxo6*^−/−^ cells were found to form typical NSC colonies in EGF/FGF-2, with a small but insignificant decrease in colony number compared to parental cells (Fig. 3E). This indicated that FOXO6 is not necessary for NSC proliferation or colony formation under optimal self-renewing conditions.

Following BMP4 treatment at low density for 24 h, both parental and *Foxo6*^−/−^ NSCs displayed a characteristic change to a stellate astrocytic morphology (Fig. 3F). Immunocytochemistry (ICC) confirmed a decrease in NES and increase in GFAP expression in parental and *Foxo6*^−/−^ NSCs after 3 days of BMP4 treatment (Fig. 3G). qRT-PCR analyses showed upregulation of astrocytic/quiescence markers (*Gfap*, *Aqp4*, *Id1*, *Cd9*) and downregulation of NSC and cell cycle markers [*Nes*, *Olig2*, *Egfr*, *cMyc* (also known as *Myc*), *Plk1*, *Cdk4*] in parental and *Foxo6*^−/−^ cells (Fig. 3H). Cell cycle exit following BMP4 treatment in parental and *Foxo6*^−/−^ cells was confirmed by loss of EdU incorporation (Fig. 3I). Proliferation and quiescence entry analyses in additional *Foxo6*^−/−^ clonal lines showed consistent results, with only one out of three knockout (KO) lines displaying altered proliferation (Fig. S1C-E). These results suggest that NSC identity is not lost following *Foxo6* disruption, and that *Foxo6* is not required for cytostatic BMP response and entry into the quiescent state.

**Fig. 4.**
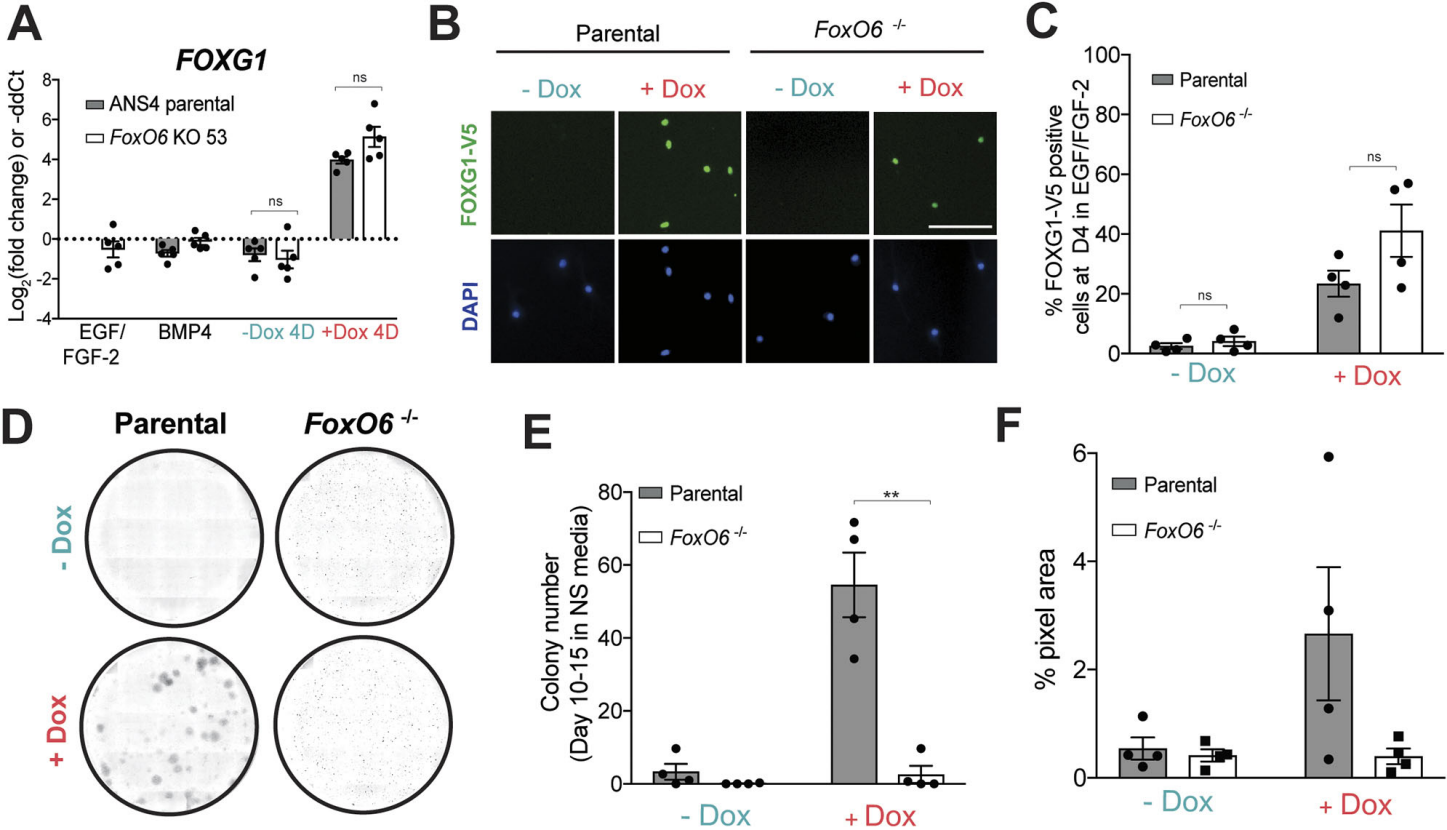
**FOXG1-induced reactivation of quiescent NSCs is inhibited upon *Foxo6* loss.** (A) qRT-PCR analysis of *FOXG1* transgene expression in parental or FOXO6 KO 53 cells engineered with inducible FOXG1-V5 after 24 h BMP4 and return to EGF/FGF-2 with or without Dox for 4 days. Expression shown relative to parental non-BMP treated (EGF/FGF-2) control [in which log_2_(fold change)=0 (dotted line)]. Mean±s.e.m., *n*=5 independent experiments. Each data point shows the mean of one experiment performed in technical duplicates. Two-tailed paired Student's *t*-test. ns, not significant. (B) Representative ICC images showing FOXG1-V5 expression after 24 h BMP4 treatment and 4 days in EGF/FGF-2, with or without Dox, in parental and FOXO6 KO 53 cells with inducible FOXG1-V5. Scale bar: 100 μm. (C) Percentage of parental or FOXO6 KO 53 cells with inducible FOXG1 expressing FOXG1-V5 (assessed by ICC) after 24 h BMP4 treatment and 4 days in EGF/FGF-2, with or without Dox. Mean±s.e.m., *n*=4 independent experiments. Each data point shows the mean of one experiment performed in technical triplicates. Two-tailed paired Student's *t*-test. ns, not significant. (D) Representative images of colony formation assay with parental and FOXO6 KO 53 cells at day 10 in EGF/FGF-2, with or without Dox. Plates stained with Methylene Blue following fixation. (E) Numbers of colonies formed after 24 h BMP4 treatment and 10-15 days in EGF/FGF-2, with or without Dox as in D. Mean±s.e.m., *n*=4 independent experiments. Each data point shows the mean of one experiment performed in technical triplicates. Two-tailed paired Student's *t*-test. **P≤0.01. (F) Percentage of the well area covered by cells after 24 h BMP4 treatment and 10-15 days in EGF/FGF-2, with or without Dox as in D. Mean±s.e.m., *n*=4 independent experiments. Each data point shows the mean of one experiment performed in technical triplicates.

We next tested whether FOXO6 was essential for exit from quiescence. Parental and *Foxo6*^−/−^ NSCs were transfected with the Dox-inducible FOXG1-V5 overexpression construct using the PiggyBac transposase system. Quiescence exit was assessed following BMP4 treatment and return of cells to EGF/FGF-2 for 10 days, with or without FOXG1-V5 induction (−/+ Dox), as previously mentioned. Similar levels of transgene induction were achieved in parental and *Foxo6*^−/−^ populations with inducible FOXG1, as assessed by FOXG1-V5 qRT-PCR and ICC (Fig. 4A-C). Strikingly, *Foxo6*^−/−^ cells showed markedly reduced capacity to reform proliferative NSC colonies (Fig. 4D-F) and, unlike the parental cells, did not highly upregulate the NSC marker NES or proliferative marker Ki67 upon FOXG1 induction (Fig. S2A). *Foxo6*^−/−^ NSCs showed striking morphological changes from the typical ‘fried-egg’, stellate astrocytic morphology, to an elongated spindle shape that was distinct from the typical bipolar phase-bright morphology of parental cells with FOXG1 induction (Fig. S2B). After an extended period (25 days), only minor evidence of colony formation was seen in the *Foxo6*^−/−^ population (Fig. S2C). Together, these data suggest that *Foxo6* is a key downstream effector of elevated FOXG1, required for efficient transition from quiescence to proliferation. Without FOXO6, quiescent cells fail to undergo the shape changes and cell cycle re-entry typical of quiescence exit in NSCs.

### Elevated FOXO6 induces the formation of large acidic vacuoles by macropinocytosis

**Fig. 5.**
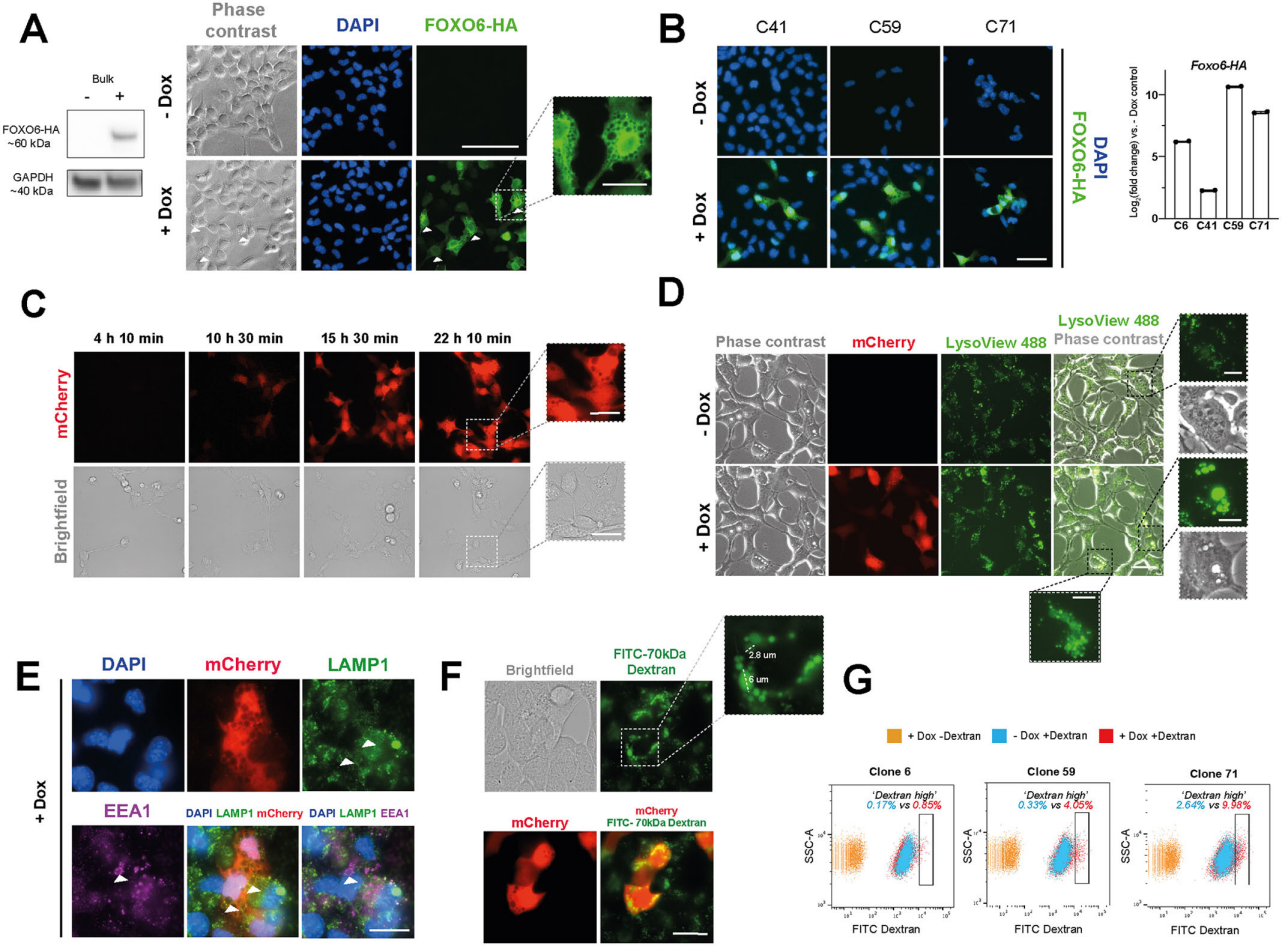
**Elevated FOXO6 induces the formation of large acidic vacuoles by macropinocytosis.** (A) Western blotting (left) and ICC (right) confirming expression of FOXO6-HA transgene following 24 h Dox treatment. Image inset highlights the appearance of vacuoles in FOXO6-HA-overexpressing cells (+Dox). Scale bars: 100 μm or 25 μm (inset). GAPDH is used as a housekeeping loading control. (B) Left: ICC of FOXO6-HA overexpression showing vacuolisation upon Dox addition to clonal NSC lines with Dox-inducible *Foxo6-HA-IRES-mCherry* expression. Scale bar: 50 μm. Right: qRT-PCR for FOXO6-HA expression in clonal cell lines (mean±s.d., technical duplicates; −Dox=0 for each clonal line). (C) Live imaging following Dox addition to clonal NSCs with Dox-inducible *Foxo6-HA-IRES-mCherry* expression (C71). Dox was added 4 h prior to imaging. Images were obtained every 10 min for ∼18 h. Scale bars: 100 μm or 25 μm (insets). (D) Imaging of LysoView-488 accumulation in clonal FoxO6-inducible cell line (C71) with or without Dox addition (2 days). Scale bars: 50 μm or 10 μm (insets). (E) ICC for lysosomal marker LAMP1 and early endosomal marker EEA1 to visualise colocalisation with vacuoles (arrowheads) (C59, following overnight Dox incubation). Scale bar: 25 μm. (F) Live imaging of 70 kDa FITC-dextran uptake and mCherry expression following incubation with Dox overnight in FOXO6-inducible cell line (C59). Scale bar: 25 μm. (G) Flow cytometry-based quantification of 70 kDa FITC-dextran uptake following incubation with Dox overnight in FOXO6-inducible cell lines (6, 59, 71). Samples displayed are +Dox −Dextran control (orange), −Dox +Dextran (blue) and +Dox +Dextran (red). Gating shows ‘Dextran high’ population. Percentages represent the increase in ‘Dextran high’ cells upon Dox addition.

To explore the specific pathways through which FOXO6 might operate to stimulate quiescence exit, we next tested the effects of forced FOXO6 expression. We established adult mouse NSCs with Dox-inducible *Foxo6-HA-IRES-mCherry* overexpression using the PiggyBac transposase system. Following fluorescence-activated cell sorting enrichment of mCherry-positive cells, FOXO6-HA overexpression was confirmed by western blotting, ICC and qRT-PCR analysis (Fig. 5A,B).

We used these transfected cells to explore cellular responses to elevated FOXO6 by microscopy. Strikingly, prominent vacuolisation was observed upon FOXO6 overexpression across multiple clonal lines (Fig. 5A,B). Live cell imaging across a time course revealed vacuole formation occurred within 10-11 h of Dox addition, coincident with mCherry expression (Fig. 5C; Movie 1). Neither treatment of untransfected adult mouse NSCs with Dox nor overexpression of an alternative transcription factor (using the same plasmid constructs with only the gene of interest substituted) resulted in vacuole formation, indicating that the phenotype was specific to FOXO6 overexpression and not a result of Dox treatment, the HA tag or mCherry overexpression (Fig. S3A,B).

We next characterised the resulting vacuoles using various imaging methods. We first ruled out these structures being lipid droplets or enlarged lysosomes, which have been implicated in quiescence regulation (Leeman et al., 2018; Ramosaj et al., 2021). Staining with the neutral lipid dye BODIPY did not reveal colocalisation with the vacuoles (Fig. S3C). In contrast, following LysoView™ incubation, we observed strong fluorescent signal colocalised with the vacuoles, indicating their acidification (Fig. 5D). Interestingly, not all structures showed equal LysoView™ accumulation, suggesting that they were at different stages of acidification and maturation, and therefore not simply enlarged lysosomes. Consistently, ICC for the lysosomal membrane marker, LAMP1, and the early endosomal marker, EEA1, did not reveal uniform colocalisation with the vacuoles (Fig. 5E), and western blot analysis showed no clear increase in EEA1 or LAMP1 expression upon FOXO6-HA induction (Fig. S3E).

Uptake of a fluorescent EGF ligand revealed much smaller puncta, indicating that the vacuoles were not formed by receptor-mediated endocytosis (Fig. S3D). Instead, the vacuole size [as large as 6 μm in diameter (Fig. 5F)] was strongly suggestive of non-selective macropinocytosis – an actin-driven process by which extracellular contents are engulfed and processed along the endosomal pathway (Swanson and Watts, 1995; Kerr and Teasdale, 2009; Lim and Gleeson, 2011). To test this hypothesis, we incubated cells with high-molecular-mass 70 kDa FITC-dextran, a well-established marker of macropinocytosis, the large size of which makes it incompatible with uptake by smaller endocytic vesicles (Commisso et al., 2014; Galenkamp et al., 2019). In cells treated overnight with Dox, clear FITC-dextran uptake was visible within the vacuoles (Fig. 5F). Flow cytometry quantification of FITC-dextran uptake confirmed an increase in the percentage of ‘Dextran high’ cells following Dox addition compared to −Dox controls across three FOXO6-HA inducible cell lines (Fig. 5G; Fig. S3F).

EdU analysis suggested that vacuolisation did not provide a growth advantage; instead, highly vacuolated cells were associated with EdU negativity following a 24 h pulse, where EdU was incorporated into newly synthesised DNA during this period as a measure of cycling cells (Fig. S3G,H). The vacuolated cells could be passaged and remained in culture (Fig. S3I), ruling out a novel form of cell death induced by hyperactivated macropinocytosis in cancer named methuosis (Overmeyer et al., 2008; Song et al., 2021). Together, these observations suggest that macropinocytosis, or the pathways that stimulate it, are an important feature of FOXO6 activity as cells exit quiescence. Macropinocytosis in cancer is associated with nutrient acquisition to aid proliferation (Commisso et al., 2013; Recouvreux and Commisso, 2017). The lack of proliferative advantage conferred by FOXO6 overexpression is consistent with the need for other supporting pathways downstream of FOXG1 for quiescence exit.

### Pak1 expression is upregulated upon FOXG1 elevation and downregulated upon FOXO6 loss in proliferative NSCs

The p21 (Cdc42/Rac)-activated kinase, Pak1, is a specific regulator of macropinocytosis controlling actin cytoskeleton dynamics (Dharmawardhane et al., 2000), and has been reported as being a FOXO6 target in the transcriptional pathway orchestrating neuronal polarity (De La Torre-Ubieta et al., 2010). Both FOXO6 and Pak1 have published roles in memory consolidation and synaptic function (Salih et al., 2012; Civiero and Greggio, 2018).

**Fig. 6.**
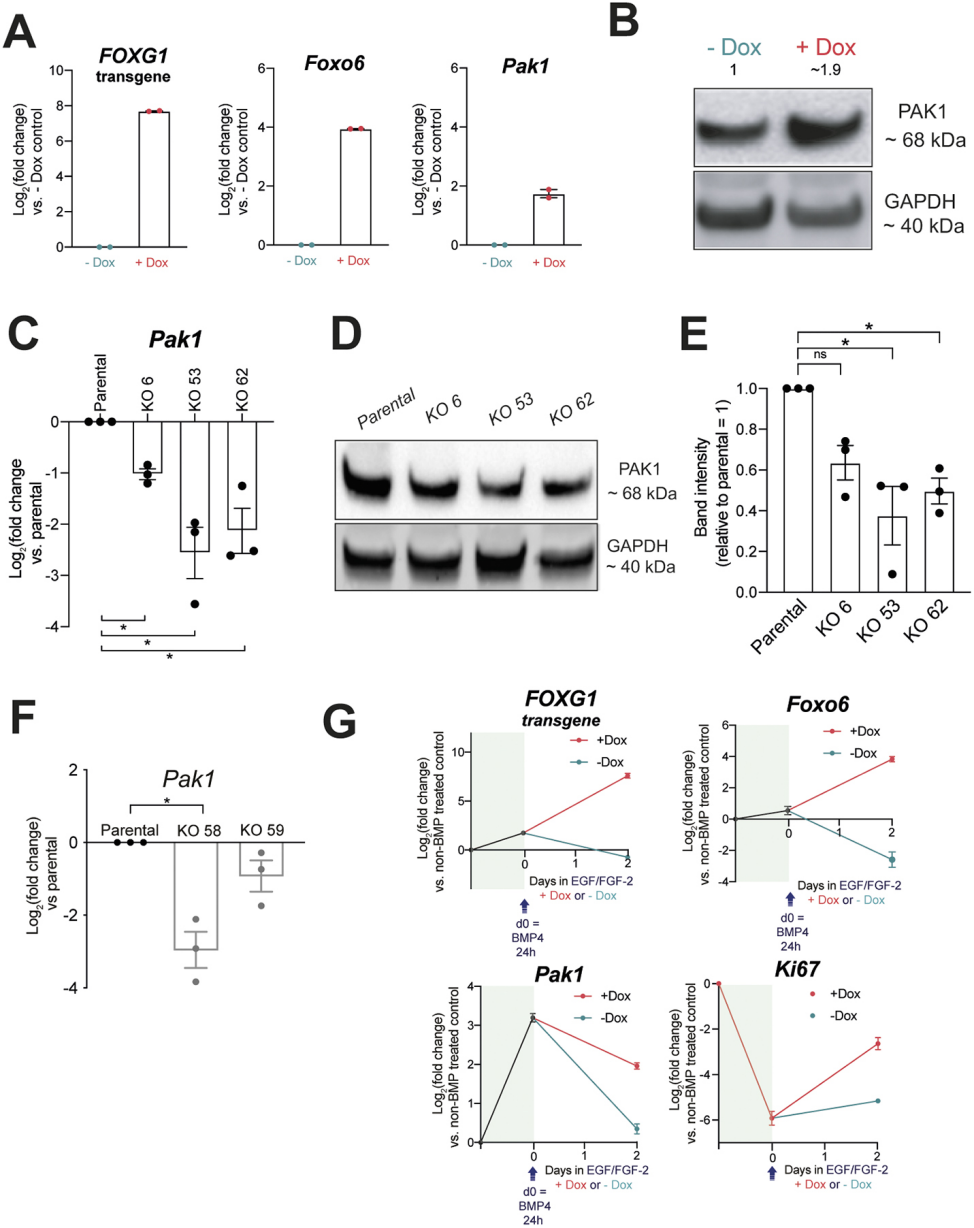
**Pak1 expression is upregulated upon FOXG1 elevation and downregulated upon FOXO6 loss in proliferative NSCs.** (A) qRT-PCR analysis of *FOXG1* transgene, and endogenous *Foxo6* and *Pak1* expression in F6 cells with Dox-inducible FOXG1-V5 grown in EGF/FGF-2 for 24 h with or without Dox (*n*=2 biological replicates, mean±s.e.m.; each data point shows the mean of one experiment performed in technical duplicates). (B) Western blot analysis of Pak1 expression in F6 cells treated with Dox in EGF/FGF-2 for 24 h. GAPDH is used as a loading control. Quantification of Pak1 bands normalised to GAPDH and −Dox control shown, where −Dox=1. (C) qRT-PCR analysis of *Pak1* expression in ANS4 parental versus FOXO6 KO clones 6, 53 and 62 (*n*=3 biological replicates, mean±s.e.m.; each data point shows the mean of one experiment performed in technical duplicates). Two-tailed one-sample *t*-test. *P<0.05. (D) Western blot analysis of Pak1 expression in parental versus FOXO6 KO clones 6, 53 and 62 (*n*=3 biological replicates). (E) Quantification of Pak1 western blot band intensities as in D. Parental=1 (*n*=3 biological replicates, mean±s.e.m.; each data point shows the intensity from one experiment). Two-tailed one-sample *t*-test. ns, not significant. *P<0.05. (F) qRT-PCR analysis of *Pak1* expression in IENS-GFP *Foxg1* KO clonal lines, compared to parental IENS-GFP [in which log_2_(fold change)=0]. Mean±s.e.m., *n*=3 independent experiments. Each data point shows the mean of one experiment performed in technical duplicates. Two-tailed one-sample *t*-test. *P≤0.05. (G) qRT-PCR analysis of *FOXG1* transgene, and endogenous *Foxo6*, *Pak1* and *Ki67* expression in F6 cells after 24 h BMP4 treatment and return to EGF/FGF-2 with or without Dox for 2 days. Expression shown relative to non-BMP-treated (EGF/FGF-2) control [in which log_2_(fold change)=0]. d0=expression after 24 h BMP4 treatment (*n*=2 biological replicates, mean±s.e.m.; each data point shows the mean of one experiment performed in technical duplicates).

To investigate a potential involvement of Pak1, we explored whether its levels were modulated by FOXG1 or FOXO6. qRT-PCR and western blot analysis revealed an increase in Pak1 expression in proliferating NSCs (compared to the −Dox control) upon FOXG1 induction (Fig. 6A,B). Furthermore, Pak1 expression was shown to be decreased in *Foxo6*^−/−^ proliferative NSCs by both qRT-PCR and western blot analysis (Fig. 6C-E). Pak1 expression was also decreased in *Foxg1*-null mouse GBM IENS cells, which have significantly reduced *Foxo6* expression as shown in Fig. 1H (Fig. 6F). This suggests that, in proliferative culture conditions, FOXO6 is needed to sustain Pak1 expression, consistent with a potentially important role in the earliest phases of cell cycle re-entry during quiescence exit. Finally, qRT-PCR analysis of *FOXG1*, *Foxo6* and *Pak1* following FOXG1-induced quiescence exit (in F6 cells) revealed higher levels of *Pak1* in +Dox compared to the −Dox control, coincident with *FOXG1* inducing *Foxo6* upregulation (Fig. 6F,G). In summary, our findings show that FOXG1-mediated induction of FOXO6 is required for efficient quiescence exit of NSCs. The link between FOXG1/FOXO6 and Pak1 is an interesting avenue of further exploration, whereby modulation of a Pak1-related pathway as cells undergo regulatory changes (such as shape and nutrient requirements) could aid preparation for cell cycle re-entry.

## DISCUSSION

Understanding the molecular mechanisms governing control of NSC quiescence has important implications in GBM, a highly aggressive adult brain cancer in which quiescent NSC-like stem cells drive relapse. Our findings here extend our previous observations that high levels of FOXG1 and SOX2 drive a proliferative radial glial-like NSC phenotype, in part through repression of the tumour suppressor *Foxo3* (Bulstrode et al., 2017). Here, we show that *Foxo6*, an underexplored FOXO member, is a downstream target activated by elevated FOXG1.

Although FOXO3 has been well described as a tumour suppressor that preserves NSC quiescence (Renault et al., 2009; Liu et al., 2018), our data suggest that FOXO6 has an opposite, pro-proliferative, role in FOXG1-induced quiescence exit of NSCs. *Foxo6* loss did not impair NSC proliferation or BMP4-induced quiescence entry. We observed NSC and cell cycle marker downregulation, astrocytic/quiescence marker upregulation, morphological changes and cell cycle exit upon BMP4 treatment, all indicating entry into a quiescent state (Codega et al., 2014; Dulken et al., 2017; Llorens-Bobadilla et al., 2015). However, FOXO6 loss was found to significantly impair FOXG1-induced exit from quiescence. Elevated FOXO6 has indeed been associated with stimulating proliferation and progression in several cancers (Qinyu et al., 2013; Rothenberg et al., 2015; Wang et al., 2017; Lallemand et al., 2018). FOXO6 has also been reported to transcriptionally control SOX2, STAT3 and Hippo signalling, all reported to control NSC and GSC self-renewal or proliferation (Salih et al., 2012; Rothenberg et al., 2015; Yang et al., 2016; Bulstrode et al., 2017; Ganguly et al., 2018; Sun et al., 2018).

Our functional studies of FOXO6 suggested that forced expression alone will trigger macropinocytosis – a process involving Pak1-regulated actin cytoskeleton remodelling. Together with literature on both FOXO6 and Pak1 in neuronal polarity and synaptic function (De La Torre-Ubieta et al., 2010; Salih et al., 2012; Civiero and Greggio, 2018), this led us to investigate Pak1 levels in relation to FOXG1 and FOXO6. Our data suggest that, in proliferating NSCs (with mitogens EGF and FGF-2), FOXO6 is required to sustain Pak1 expression and FOXG1 induction can result in even higher PAK levels. Although this is not functionally important in sustaining NSC proliferation, as shown by the lack of proliferation defects upon *Foxg1* or *Foxo6* deletion (Bulstrode et al., 2017), these changes in Pak1 levels may impact regulatory transitions, such as cell shape and metabolic requirements, as cells prepare to exit quiescence into the proliferative radial-glia like state. Indeed, Pak1 levels are increased by BMP signalling (Fig. 6F), likely as cells undergo extensive restructuring. Our data lead us to speculate a working model in which FOXO6 is activated downstream of FOXG1, facilitating exit from quiescence. Although a direct link between Pak1 and quiescence exit has not yet been established, it is plausible that part of FOXG1/FOXO6 function could be to, directly or indirectly, trigger a Pak1-related signalling pathway that alters actin dynamics and related cell shape/nutrient-sensing pathways required for quiescence exit.

As vacuolisation was not observed upon FOXG1 overexpression (Fig. S3J), it is possible that FOXO6-induced macropinocytosis represents a stalled state, with other pathways downstream of FOXG1 necessary to be activated concomitantly to ensure cell cycle re-entry, e.g. through increased pinocytic flux that cannot be assessed within our experimental timeframes. Indeed, active Pak1 has been found to modulate pinocytic cycling, enhancing both FITC-dextran uptake and efflux (Dharmawardhane et al., 2000). It is plausible that such an enhancement in pinocytic cycling may aid rewiring of the metabolome required for the transition from quiescence to proliferation (Lee et al., 2017; Adusumilli et al., 2021; Wani et al., 2022). This will require further deeper exploration in future studies. Alternatively, hyperactivation of signalling pathways upon FOXO6 overexpression may result in macropinocytosis as a metabolic stress response. Hyperactivation of Ras signalling, canonical Wnt and PI3K signalling have all been shown to play roles in inducing macropinocytosis (Overmeyer et al., 2008; Recouvreux and Commisso, 2017; Tejeda-Muñoz et al., 2019). Interestingly, FOXG1 was recently found to synergise with Wnt signalling in driving quiescence exit in GBM (Robertson et al., 2023). The activity of FOXO factors is controlled by phosphorylation downstream of IGF/PI3K/AKT signalling (Hay, 2011; Jiramongkol and Lam, 2020). PAK1 is upregulated in various cancer types, integrates various signalling pathways, such as PI3K and RAS, and has been reported to phosphorylate and inactivate FOXO1 in breast cancer (Mazumdar et al., 2003) and FOXO6 in liver ageing (Kim et al., 2015). It is therefore also possible that FOXO6 elevation results in signalling activation that, in turn, reinforces phosphorylation and deactivation of FOXO tumour suppressors.

FOXO factors are known to modulate metabolic functions in homeostasis and cancer (Paik et al., 2007; Kim et al., 2011; Chung et al., 2013; Yadav et al., 2018). FOXO3 protects NSCs against oxidative stress and controls their glucose metabolism to ensure optimal self-renewal (Renault et al., 2009; Yeo et al., 2013), in part through *cMyc* inhibition (Peck et al., 2013). In contrast, FOXO6 promotes gastric cancer cell proliferation through *cMyc* induction (Qinyu et al., 2013), and its loss inhibits colorectal cancer cell proliferation, invasion and glycolysis, with decreased PI3K/AKT/mTOR pathway activation (Li et al., 2019). In GBM, mTORC2 signalling controls glycolytic metabolism through inhibition of FOXO1/3 and de-repression of *cMyc* (Masui et al., 2013). Elevated FOXG1, itself implicated in regulating mitochondrial functions (Pancrazi et al., 2015), may therefore alter FOXO3 and FOXO6 expression to result in deregulated energetics that drive a proliferative state and/or oppose quiescence. Macropinocytosis in cancer has been reported to aid nutrient uptake (Commisso et al., 2013; Recouvreux and Commisso, 2017); the role of FOXO6 in linking GSC state transitions with metabolism will therefore be an interesting avenue for further exploration. Although it therefore remains to be determined whether macropinocytosis upon FOXO6 overexpression is functional physiologically in quiescence exit, this observation gives interesting insights into the potential signalling downstream of this underexplored FOXO family member.

With respect to normal NSCs, the roles of FOXO6 in the developing and adult brain are less well defined than for FOXO3 (Salih et al., 2012; Sun et al., 2018). The changing spatial pattern of FOXO6 expression during mouse brain development suggests different functions at distinct stages; yet, the NSC number at embryonic day (E)18 is unchanged in *Foxo6*-null mice (Hoekman et al., 2006; Paap et al., 2016). Cortical FOXO6 levels decrease significantly after birth, with adulthood expression regulating synapse formation in the hippocampal CA1/3 regions, as well as cerebellar neuronal polarity (De La Torre-Ubieta et al., 2010; Salih et al., 2012). Like for FOXG1, the homeostatic roles of FOXO6 may therefore be subtle in adulthood, and mostly involved in neural plasticity (Yu et al., 2019). This is in keeping with our finding that basal FOXO6 levels are low in adult NSCs and not required for sustained proliferation but are important for cell state transitions. If the FOXO6 levels activated by elevated FOXG1 represent an acquired dependency of GBM, there may be a therapeutic window to target this pathway. However, given the poorly understood roles of FOXO6, further work is needed to determine its specific value as a therapeutic target. Regardless, the balance between these three FOX family members – FOXG1, FOXO3 and FOXO6 – has been revealed by our studies, and others, to be a key signalling node in the context of GBM quiescence control and warrants further investigation.

## MATERIALS AND METHODS

### Cell culture

Mouse NSC lines were derived from adult SVZ as described previously (Conti et al., 2005; Sun et al., 2008). IENS cells, described previously with *Ink4a/ARF* (also known as *Cdkn2a*) deletion and EGFRvIII overexpression (Bruggeman et al., 2007; Bulstrode et al., 2017), were kindly provided by Professor M. Van Lohuizen (Netherlands Cancer Institute, Amsterdam, The Netherlands). Established lines were cultured in an adherent monolayer on uncoated tissue culture plastics, at 37°C with 5% CO_2_, with serum-free ‘complete’ NSC medium. This medium consists of Dulbecco's modified Eagle medium/HAMS-F12 (Sigma-Aldrich, D8437) supplemented with N2 and B27 (Life Technologies/Gibco), penicillin, streptomycin (Gibco), bovine serum albumin (Gibco), β-mercaptoethanol (Gibco), minimum essential medium non-essential amino acids (Gibco), 1 μg/ml laminin (Sigma-Aldrich or Cultrex), 10 ng/ml mouse EGF and 10 ng/ml human FGF-2 (Peprotech). Medium was exchanged every 3-4 days. Cell lines were routinely confirmed as *Mycoplasma* negative. Cells were dissociated once 70-80% confluency was reached using Accutase solution (Sigma-Aldrich), passaged approximately 1:6 every 3-4 days. Quiescence was induced by plating cells at a density of 10 cells/mm^2^ in NSC medium in the absence of EGF/FGF-2 and supplemented with BMP4 (10 ng/ml, Peprotech). Cells were treated for 1 day or 3 days, as indicated.

### Derivation of genetically engineered cell lines

F6 and F11-19 cell lines were derived previously (Bulstrode et al., 2017). Stable transgene integration using the PiggyBac system was used to derive bulk populations of parental ANS4 and *Foxo6*^−/−^ mouse NSCs with Dox-inducible *FOXG1-V5* overexpression. Cells were transfected using the Amaxa 4D nucleofection system (Lonza) in 16-well cuvette strips, using the DN100 programme. Cells (4×10^5^) were transfected in 20 μl SG cell line transfection buffer with a total of 800 ng DNA, consisting of the CMV-PiggyBac transposase vector (PBase), pCAG-Tet3G vector (encoding the Tet-On 3G transactivator protein, rtTA) and the TetOn *FOXG1-V5* expression vector in a 2:1:1 ratio. Following recovery, Dox was added (1000 ng/ml) for 24 h. Selection for *FOXG1-V5* expression cassette integration was then commenced by supplementing NSC medium with Dox and blasticidin (5 μg/ml). All mock transfected control cells died within 7 days of selection. The surviving transfected population was then expanded in NSC medium, and Dox-inducible *FOXG1-V5* expression was confirmed by ICC and qRT-PCR. Cells were reselected for stable transgene expression between independent experiments. The resulting population was expanded for 3-4 days in NSC medium prior to functional assays, during which time existing FOXG1-V5 protein was degraded. Stable transgene integration using the PiggyBac system was also used to derive ANS4 cells with Dox-inducible FOXO6-HA-IRES-mCherry overexpression. The TetOn FOXO6-HA-IRES-mCherry vector was derived using the extensible mammalian modular assembly toolkit (EMMA) system (Martella et al., 2017). All EMMA parts are sequence verified, including the *Foxo6* coding sequence ordered from GeneArt Gene Synthesis (Thermo Fisher Scientific).

For CRISPR/Cas9-mediated gene KO of *Foxg1* in mouse IENS-GFP, cells were transfected using the Amaxa 4D nucleofection system (Lonza) and the DN100 programme. Cells (1.5 million) were transfected in 100 μl SG cell line transfection buffer with a total of 4 μg DNA, consisting of 2 μg wild-type Cas9-2A-mCherry vector and 1 μg of each single-guide RNA (sgRNA) plasmid. For sgRNA-encoding plasmids, single-stranded oligonucleotides (IDT) containing the guide sequence of the sgRNAs were annealed, phosphorylated and ligated into BsaI site of U6-BsaI-sgRNA backbone (kindly provided by S. Gerety, Sanger Institute, Cambridge, UK). Three days post-transfection, Cas9-mCherry-expressing cells were isolated by fluorescence-activated cell sorting. Loss of FOXG1 was confirmed and the transfection efficiency was estimated in the bulk sorted population by ICC. For derivation of clonal cell lines, 300 cells were plated per 10 cm dish. After 10-15 days, discrete colonies were picked, expanded and screened for successful disruption of *Foxg1* by PCR genotyping and ICC. Loss of FOXG1 protein expression was validated by western blotting.

CRISPR/Cas9-mediated gene KO of *Foxo6* in ANS4 cells was performed using a strategy described in Bressan et al. (2017), using two sgRNAs targeting the *Foxo6* exon, Cas9 nickase and a targeting vector comprising an *EF1a*-puromycin antibiotic resistance cassette flanked by 1 kb homology arms specific for the locus. Parental ANS4 cells were transfected using the Amaxa 2B nucleofection system (Lonza). CRISPR/Cas9-mediated HA tagging of FOXO6 was performed using the Cas9 ribonucleoprotein single-stranded oligodeoxynucleotide strategy described in Dewari et al. (2018). Once recovered, cells were assessed for successful tag integration by PCR genotyping, ICC and western blotting. See Table S1 for all CRISPR/Cas9 gene editing sequences.

### PCR-based genotyping of genetically engineered cell lines

Genomic DNA (gDNA) isolation from bulk transfected cells and clonal cell lines was performed using a DNeasy Blood and Tissue kit (Qiagen), according to the manufacturer's protocol. DNA concentrations were quantified using a NanoDrop™ spectrophotometer (Thermo Fisher Scientific). All primers were designed using Primer3 software. To identify non-homologous end joining-based indel formation, the region flanking the guide RNA (gRNA) target site was amplified using gene-specific primers. In case of *Foxg1* deletion from IENS-GFP, primers were designed flanking the 5′ and 3′ gRNA targeting sites. For validation of *Foxo6* gene deletion, primers were designed as described in Bressan et al. (2017) (PCR1, 2 and 3). For validation of HA tag knock-in at the *Foxo6* locus, primers were designed flanking the tag, outside of the 77[Supplementary-material sup1] bp 5′ and 3′ homology arms. PCR products were analysed using 1-2.5% agarose gels with ethidium bromide and GeneRuler™ 1 kB plus DNA ladder (Thermo Fisher Scientific). Gels were imaged on a UV gel reader or Bio-Rad ChemiDoc™ Imager. See Table S2 for all primer sequences.

### ICC

Cells were fixed in 4% paraformaldehyde (PFA) for 10 min, permeabilised in PBS with 0.1% Triton X-100 and blocked in 0.1% bovine serum albumin plus 3% goat serum solution for 1 h at room temperature. Samples were incubated overnight with primary antibodies at 4°C followed by incubation with appropriate secondary antibodies (1:1000; Invitrogen, Alexa Fluor™ 488/594/647) for 1 h at room temperature. Cells were incubated in 4′,6-diamidino-2-phenylindole (DAPI; 1:10,000) for 5 min for nuclear counterstaining. Imaging was performed using a Nikon TiE microscope and NIS software. Analysis was performed using FIJI (ImageJ) software. Quantification of immunopositive cells was performed using the Cell Counter plugin. Total cell number was determined by DAPI staining. Quantification of FOXG1-V5 staining was performed using PerkinElmer's Operetta High-Content Imaging System and Columbus software. The following primary antibodies were used: anti-NES (1:10; Developmental Studies Hybridoma Bank, Rat-401), anti-GFAP (1:1000; Sigma-Aldrich, G3893), anti-FOXG1 (1:100; homemade 17B12 hybridoma, Pollard laboratory), anti-V5 tag (1:2000; eBioscience, 14-6796), anti-HA tag (1:100; Cell Signaling Technology, 6E2 2367), anti-Ki67 (1:200; Thermo Fisher Scientific, RB-9043-P0), anti-EEA1 (1:200; Cell Signaling Technology, 3288) and anti-LAMP1 (1:600; Abcam, ab25245).

### Western blotting

Immunoblotting was performed using standard protocols. Membranes were blocked in 5% milk in TBS-T (TBS+0.1% Tween-20) for 1 h at room temperature and incubated with primary antibody dilutions in 5% milk in TBS-T overnight with rocking. Protein detection was carried out with horseradish peroxidase-coupled secondary antibodies. Membranes were developed using homemade enhanced chemiluminescence (ECL) solution or Clarity ECL Western Blotting Substrate (Bio-Rad) and imaged using X-ray films or a Bio-Rad ChemiDoc™ Imager. The following primary antibodies were used: anti-V5 tag (1:1000; eBioscience, 14-6796, RRID:AB_10718239), anti-FOXG1 (1:1000; homemade 17B12 hybridoma, Pollard laboratory), anti-GAPDH (1:1000; GenTex, GTX627408, RRID: AB_11174761), anti-HA tag (1:1000; Cell Signaling Technology, 6E2 2367, RRID:AB_10691311), anti-EEA1 (1:1000; Cell Signaling Technology, 3288, RRID:AB_2096811), anti-LAMP1 (1:1000; Abcam, ab25245, RRID:AB_449893) and anti-Pak1 (1:1000; Cell Signaling Technology, 2602, RRID:330222). Western blot quantification was performed in FIJI software, normalising to GAPDH loading control.

### qRT-PCR

RNA extraction was performed using a Masterpure™ RNA purification kit (Epicentre) according to the manufacturer's instructions. DNase digestion was performed using RQ1 RNase-free DNase (Promega) or Masterpure™ RNase-free DNase I (Epicentre). RNA concentration was determined using a Qubit™ RNA High Sensitivity Kit (Thermo Fisher Scientific) or NanoDrop™ Spectrophotometer (Thermo Fisher Scientific). Within each experiment, the same amount of RNA was inputted for cDNA synthesis. Reverse transcription was performed using Invitrogen Superscript III. qRT-PCR was performed using TaqMan Universal PCR Master Mix (Applied Biosystems) and TaqMan gene expression assays (Life Technologies) on a QuantStudio™7 Flex Real-Time PCR machine. No reverse transcriptase and water controls were run on each plate to ensure the absence of contamination. Technical replicates were run to ensure pipetting accuracy. Data were analysed using the ddCt method; this method assumes 100% PCR efficiency, which is guaranteed with TaqMan assays. Replicate Ct values were averaged and normalised to the housekeeping gene, *Gapdh* (to give dCt). These values were then normalised to a calibrator sample (to give ddCt). Data are presented as log_2_(fold change) or −ddCt, where this value equals zero for the calibrator, as indicated in the figure legends. The following TaqMan assays (Life Technologies) were used: hFOXG1 (Hs01850784_s1), mGapdh (Mm99999915_g1), mFoxO6 (Mm00809934_s1), mPlk1 (Mm00440924_g1), mNestin (Mm00450205_m1), mOlig2 (Mm01210556_m1), mAqp4 (Mm00802131_m1), mGfap (Mm01253033_m1), mCdk4 (Mm00726334_s1), mMyc (Mm00487804_m1), mEgfr (Mm00433023_m1), mId1 (Mm00775963), mCd9 (Mm00514275_g1) and mPak1 (Mm00817699_m1).

### Cell proliferation assays

Confluence analysis and growth curves were determined using an IncuCyte™ live cell imaging system (Essen Bioscience). Cells were plated at ∼25 cells/mm^2^ in NSC medium (EGF/FGF-2) in triplicate wells and imaged periodically until confluence was reached. For analysis of proliferation rates, cells were incubated in NSC medium (EGF/FGF-2), supplemented with 10 μM EdU for 24 h. Cells were then fixed in 4% PFA for 10 min at room temperature and stained with a Click-iT EdU Alexa Fluor 647 assay kit (Life Technologies) according to the manufacturer's instructions. Imaging was performed using the Nikon TiE microscope and NIS software. For each condition, triplicate wells were analysed (4×4 10× stitched images per well). The total cell number was determined by DAPI staining. Quantification was performed using the Image Thresholding and Particle Analysis functions on FIJI software.

### Colony formation assays

Colony formation in NSC medium (EGF/FGF-2) was assessed by plating cells at a density of 1 cell/mm^2^ (1000 cells per well of a six-well plate, with six replicate wells). Medium was changed every 3-4 days. Following 10 days, plates were fixed using 4% PFA for 10 min at room temperature. Colonies were stained using Methylene Blue for 30 min. Plates were washed gently with deionised water and allowed to dry. Plates were then imaged on a Celigo™ Image Cytometer (Nexcelom Bioscience). Colonies were counted manually using the Cell Counter plugin on FIJI, or the percentage pixel area of the well covered by colonies was quantified using FIJI Image Thresholding and Particle Analysis functions.

For assessment of colony formation following BMP4 treatment, cells with Dox-inducible FOXG1-V5 overexpression were plated at a density of 10 cells/mm^2^ (10,000 cells per well of a six-well plate), in NSC medium in the absence of EGF/FGF-2 and supplemented with BMP4 (10 ng/ml) (Bulstrode et al., 2017). After 24 h, medium was replaced fully with NSC medium containing EGF/FGF-2 with or without Dox (1000 ng/ml). Medium was then replaced every 3-4 days. Following 10-12 days, plates were fixed using 4% PFA for 10 min at room temperature. Colonies were stained using Methylene Blue for 30 min and imaged on a Celigo™ Image Cytometer (Nexcelom Bioscience). Colonies were counted manually using the Cell Counter plugin on FIJI, or the percentage pixel area of the well covered by colonies was quantified using FIJI Image Thresholding and Particle Analysis functions. Three technical replicates were averaged to give the mean number of colonies per biological replicate.

### Imaging analysis of vacuoles

Live imaging following FOXO6-HA induction was performed using the Nikon TiE microscope. Imaging began 4 h after Dox addition, and images were obtained every 10 min for ∼18 h. For lysosome assessment, 1000× LysoView488™ stock solution (Biotium, 70057) was diluted to 1× in NSC medium. Cells were incubated in medium containing 1× LysoView488™ for 30 min at 37°C prior to imaging. For lipid droplet assessment, BODIPY 493/503 (Invitrogen, D3922; 5 mg/ml) was used. For analysis of dextran uptake, 70000 MW FITC-Dextran (Invitrogen, 070621; 20 mg/ml) was diluted 1:20 to 1 mg/ml in NSC medium and added to cells overnight coincident with Dox addition if appropriate. Dextran uptake was visualised in the green channel by imaging and flow cytometry. EGF uptake was visualised by incubating vacuolated cells with medium containing 100 ng/ml EGFR ligand conjugated to a fluorophore (EGF-647; Thermo Fisher Scientific, E35351) for 1 h prior to washing and imaging.

### Statistical analyses

Statistical analyses were performed in GraphPad Prism 7. Biological replicates were considered as different passage numbers of same cell line plated in independent experiments. Mean±s.e.m. or s.d., and *n* numbers, are shown in the figure legends. Owing to small sample sizes, tests for normality and distribution were of limited value. However, this was not considered to be an impediment to parametric analysis with small *n* numbers. Statistical tests used are indicated in the figure legends. For qRT-PCR data, statistics were calculated from ddCt values. Where two-tailed one-sample *t*-tests were used, this was based on the null hypothesis that log2(fold change)/−ddCt equals zero (i.e. equal to the calibrator sample). Paired two-tailed Student’s *t*-tests were used where samples (e.g. wild-type and FOXO6 KO cells) must be matched owing to variation between biological replicates (e.g. growth analysis, colony assays following BMP4 treatment). *P*<0.05 was considered significant.

## Supplementary Material

10.1242/dmm.052005_sup1 Supplementary information
