# Supplementary material for: Modelling quiescence exit of neural stem cells reveals a FOXG1-FOXO6 axis
Source: Dis Model Mech. 2024 Nov 29;17(12):dmm052005. doi: 10.1242/dmm.052005 (PMC11625887; doi:10.1242/dmm.052005)
Supplement: Supplementary information [file dmm-17-052005-s1.pdf]

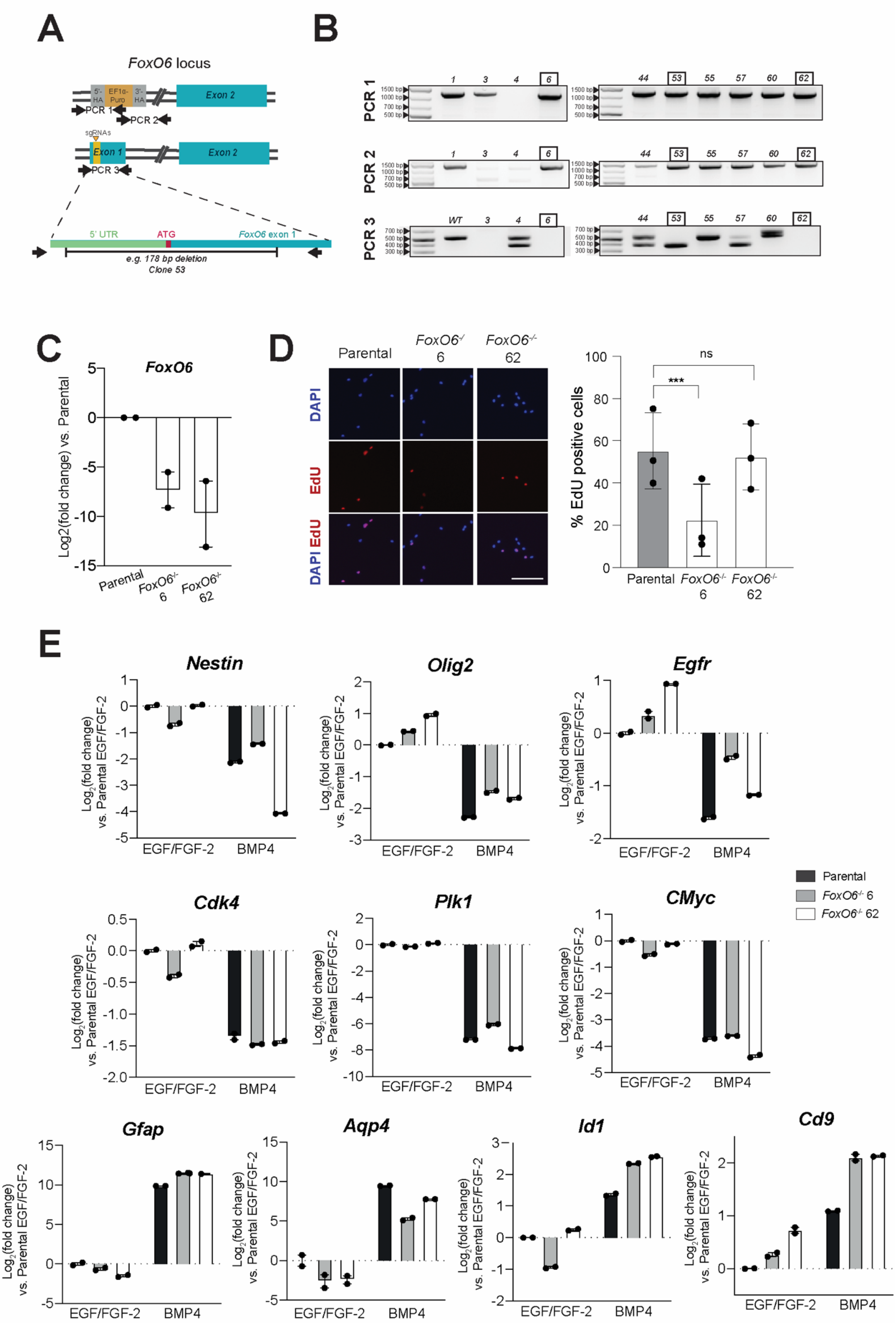
A
FoxO6 locus
Exon 2
PCR 1
PCR 2
sgRNAs
Exon 1
Exon 2
PCR 3
5' UTR
ATG
FoxO6 exon 1
e.g. 178 bp deletion
Clone 53
B
PCR 1
PCR 2
PCR 3
C
FoxO6
Log2(fold change) vs. Parental
Parental
FoxO6-/- 6
FoxO6-/- 62
D
Parental
FoxO6-/- 6
FoxO6-/- 62
DAPI
EdU
DAPI EdU
% EdU positive cells
ns
***
E
Nestin
Olig2
Egfr
Cdk4
Plk1
CMyc
Gfap
Aqp4
Id1
Cd9
Log2(fold change) vs. Parental EGF/FGF-2
EGF/FGF-2
BMP4
Parental
FoxO6-/- 6
FoxO6-/- 62

Fig. S1. FoxO6 is not essential for NSC proliferation or response to BMP4, related to Fig. 3.

(A) Schematic of *FoxO6* locus following CRISPR/Cas9-mediated knockout strategy. Yellow triangles show the sgRNA target sites, resulting in a 178 bp deletion in allele 1 in FoxO6 KO clone 53. Exon 1 of allele 2 is replaced by an EF1a-puromycin cassette.

(B) PCR genotyping of *FoxO6* KO clonal cell lines 6, 53 and 62. PCR 1 and PCR 2, across the 5' and 3' homology arms of the EF1a-puromycin cassette, respectively, show correct integration at one of the *FoxO6* alleles. PCR 3 shows a 178 bp deletion (53) or loss (6, 62) of the remaining *FoxO6* allele. WT parental band = 565 bp, knockout (53) band = 387 bp.

(C) qRT-PCR analysis of *FoxO6* mRNA levels in FoxO6 KO clonal cell lines 6 and 62, compared to parental cells (in which log_2(FC) = 0). Expression values were normalised to Gapdh. Y axis represents log_2(Fold change). Mean +/- SEM. n=3 independent experiments. Each data point shows the mean of one experiment, performed in technical duplicates.

(D) EdU incorporation assay (24h pulse) in parental and FoxO6 KO clonal lines (6 and 62) grown in EGF/FGF-2. (Left) Representative fluorescent images of EdU incorporation. Scale bar: 100 um. (Right) Plot shows mean +/- SEM, n=3 independent experiments. Each data point shows the mean of one experiment performed in technical triplicates.

(E) qRT-PCR analysis of NSC (*Nestin, Olig2, Egfr),* cell cycle marker *(Cdk4, Plk1, Cmyc)*, and astrocyte/quiescence *(Gfap, Aqp4, Id1, Cd9*) marker expression in ANS4 parental and FoxO6 KO clonal cell lines (6 and 62) in EGF/FGF-2 and after 24 h BMP4 treatment. Expression values were normalised to *Gapdh* and shown relative to the expression in parental cells in EGF/FGF-2 (in which log_2(FC) = 0, shown by the dotted line). Graph shows Mean +/- SD. One experiment, performed in technical duplicates.

Disease Models & Mechanisms • Supplementary information

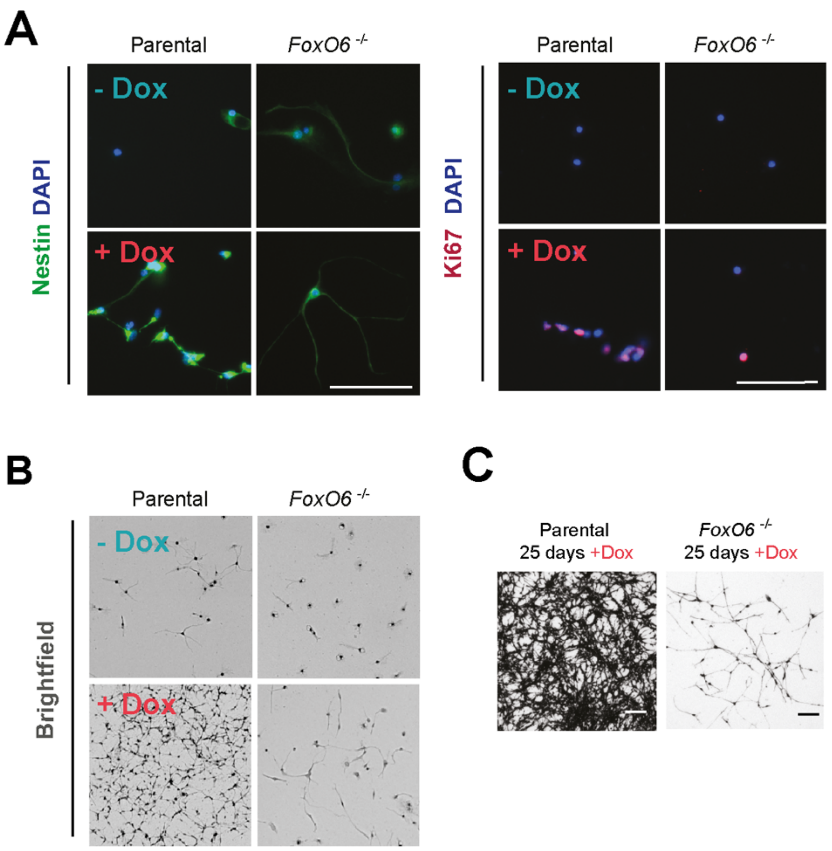

Fig. S2. FOXG1-induced reactivation of quiescent NSCs is inhibited in *FoxO6* null cells, related to Fig. 4.

(A) ICC images showing Nestin (left) and Ki67 (right) expression at Day 10 in NSC media with or without Dox addition (following 24 h BMP4 treatment) in both parental and FoxO6 $^{-/-}$ 53 cells engineered with inducible FOXG1-V5 construct.

(B) Representative brightfield images following fixation of colony assay plate and staining with methylene blue.

(C) Brightfield images of parental and FoxO6 $^{-/-}$ 53 colonies after 25 days in NSC media + Dox. Scale bars: 100 µm.

Disease Models & Mechanisms • Supplementary information

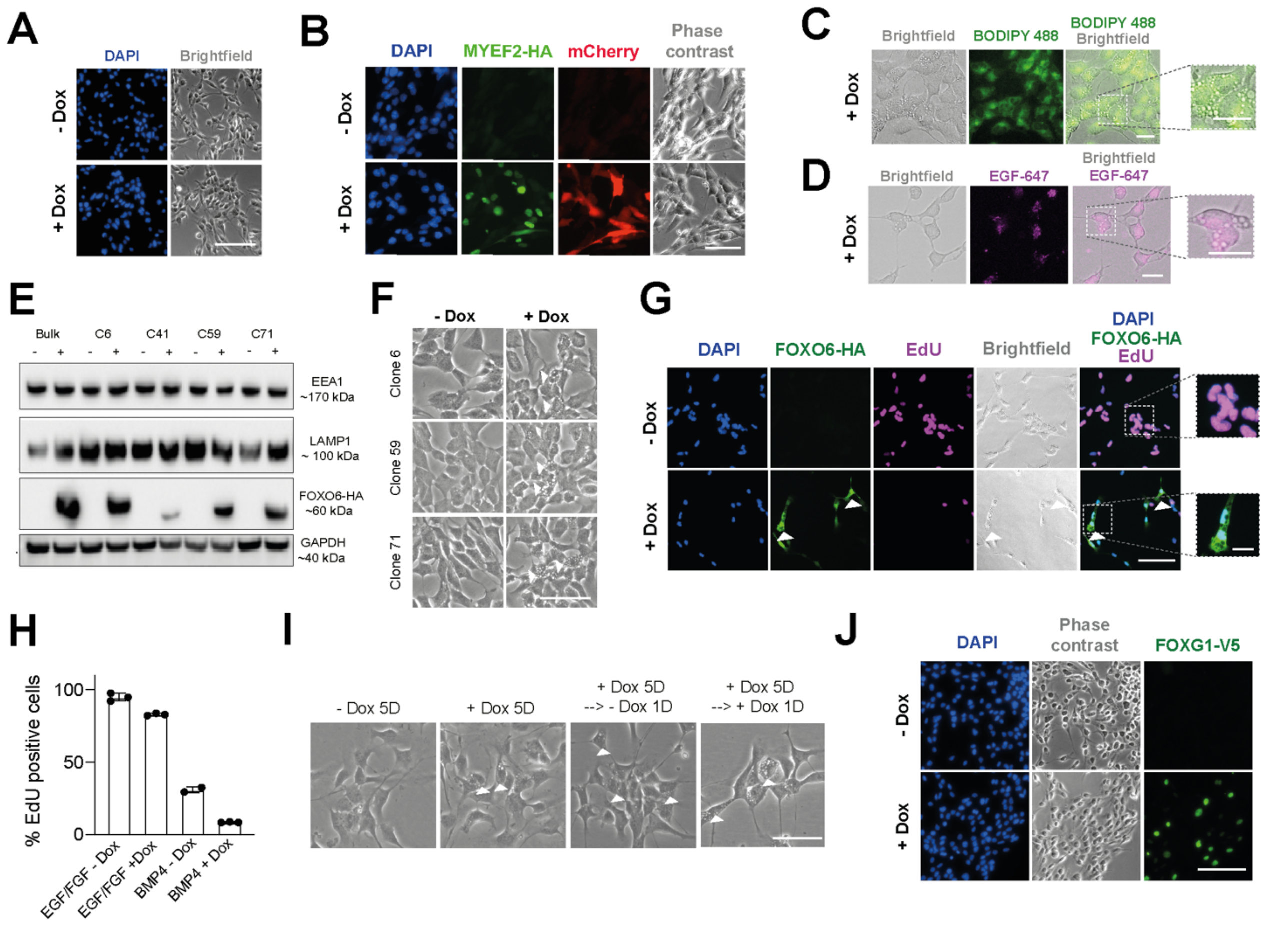

Fig. S3. Elevated FoxO6 induces the formation of large acidic vacuoles by macropinocytosis, related to Fig. 5.

(A) Phase contrast imaging following Dox addition to untransfected ANS4 cells does not induce vacuole formation. Scale bar 100 um.

(B) ICC following Dox-induced (24h) MYEF2-HA-IRES-MCHERRY overexpression in mouse GSC line ‘NPE’ shows no evidence of vacuole formation. Scale bar 50 um.

(C) BODIPY lipid staining does not colocalise with vacuole structures in FoxO6-inducible cell line (C71, 2 days +/- Dox). Scale bar 25um.

(D) EGF-647 uptake after a pulse of 1 hr shows puncta representative of receptor-mediated endocytosis (C71 incubated overnight with Dox prior to EGF-647 pulse). Scale bar 25 um.

(E) Western blot analysis of LAMP1, EEA1 and HA upon FoxO6-HA overexpression (+/- Dox). GAPDH is used as a loading control. Bulk transfected population sorted for mCherry and clonal cell lines (6, 41, 59, 71) analysed.

(F) Phase contrast images show vacuole formation in FoxO6-HA inducible cell lines following Dox+Dextran overnight incubation, prior to flow cytometry analysis. Scale bar 100 um.

(G) Imaging of EdU incorporation in FoxO6-HA inducible cells (C71) after 2 days in EGF/FGF +/- Dox (24h pulse). Scale bar 100 um or 25um.

(H) EdU incorporation after EGF/FGF-2 or BMP4 for 3 days +/- Dox (24h pulse). n=3 technical replicates, mean +/- SD.

(I) Phase-contrast images of Dox treated cells in culture (c71). Vacuolated cells remain after 5 days in Dox and following Dox removal.

(J) ICC in F6 cells with Dox-inducible FOXG1-V5 shows no evidence of vacuolisation upon Dox addition (FOXG1-V5 induction). Scale bar 100 um.

Table S1. CRISPR/Cas9 gene-editing sequences

Target gene	Purpose	Sequence type	Sequence (5' to 3')	PAM
Foxg1	Gene disruption (5')	gRNA	GACAACCACCACGCGAGCCA	CGG
Foxg1	Gene disruption (3')	gRNA	GAAATAATCAGACAGTCCCC	CGG
FoxO6	Gene disruption (gRNA 1)	gRNA	GAGGGAGGCTCCGCGGAGGG	TGG
FoxO6	Gene disruption (gRNA 2)	gRNA	GCCCGAGCCGGGCGGGACCA	TGG
FoxO6	Gene-tagging	gRNA	GGCACCCAGGCTGTGTAGGG	TGG
FoxO6	Gene-tagging	Single-stranded donor oligonucleotide	ACTTCGACTCAGCCCTGCCTCCGCCACCCCCGGGCCTGGCTGGGGCGCCGCCCCCT AACCAGAGCTGGGTGCCAGGCTACCCATACGACGTACCAGATTACGCTTGAGGGG CACCCTACACAGCCTGGGTGCCCCGGTCCCGTCCCCATGGGGCCTCTGTCTTCCCA TCCCGATCCCCGGGTCC	-

Table S2. Primers for PCR-based genotyping of genetically engineered cell lines

Locus	Modification	Forward (5' to 3')	Reverse (5' to 3')
FoxO6	C terminal (3') HA-tag	GGATCTGGACCTCGACATGT	ATCTGGTACGTCGTATGGGT
FoxO6	Gene disruption – PCR 1	CCTCCCCAACCGTTCTTAAC	GTGGTATCGTTATGCGCCTT
FoxO6	Gene disruption – PCR 2	CATGTCTGGATCCGGGGGTACCGCGTCGAG	TGTTAGGGAAGGCTTCTTGG
FoxO6	Gene disruption – PCR 3	CCTCACTGCCTGGGTCTTT	CGGACCATCCAGTCGTAGAT
FoxO6	*EF1a*-puro cassette, 5'HA amplification	AACGACGGCCAGTGAATTCGATAGCACAGTAA AAGCCCAGGA	TATCGTTATGCGCCTTGATAGGAGGCTGGACAAACTG G
FoxO6	*EF1a*-puro cassette, 3' HA amplification	CTGAGCTAGCCATCAGTGATTGGTAGGGTGAC AGGGGATA	CCATGATTACGCCAAGCTTGATTGTAGGGGACTGGAA AATGG

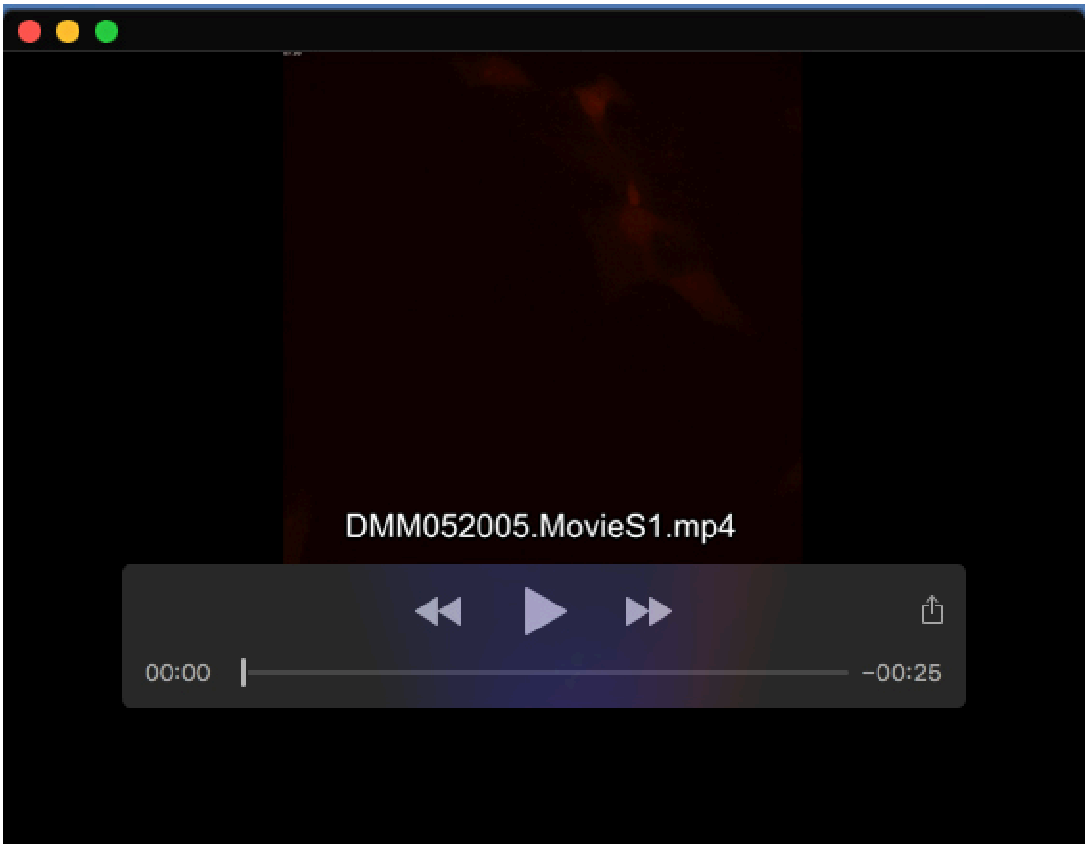

Movie 1. Live imaging following Dox addition to clonal NSCs with Dox-inducible *FoxO6-HAIRES-mCherry* expression (C71). Dox was added 4 hours prior to imaging. Images were obtained every 10 minutes for ~18 hours. Scale shown on main Fig. 5C.

Disease Models & Mechanisms • Supplementary information
